# A Multiform, Group-Based Rehabilitation Program for Visually Impaired Young People to Promote Activity and Participation. A Pilot Study

**DOI:** 10.3390/ijerph16193682

**Published:** 2019-09-30

**Authors:** Anna-Liisa Salminen, Tuija Heiskanen, Tiina Suomela-Markkanen

**Affiliations:** 1 Research department, The Social Insurance Institution of Finland, FI-00250 Helsinki, Finland; tuija.t.heiskanen@kela.fi; 2 Insurance Medicine Unit, The Social Insurance Institution, FI-40101 Jyväskylä, Finland; tiina.suomela-markkanen@kela.fi

**Keywords:** rehabilitation, activity, participation, visual impairment

## Abstract

Young people with visual impairment (YPWVI) face several challenges in their everyday lives. However, little is known about interventions that focus on promoting their participation that contributes to health and well-being and is considered the most relevant outcome in rehabilitation. Objectives: This study investigated the clinical outcomes and acceptability of a new one-year, multiform, group-based rehabilitation program for YPWVI. The aim of the pilot program was to support them becoming more independent and to promote their participation. Rehabilitation consisted of group-meetings in an institutional setting, online group meetings, individually tailored one-on-one guidance, individual online discussions and parents’ group meetings. Fifteen young persons with visual impairment were recruited and 14 completed the intervention, six of whom were blind or had severe visual impairment and eight had mild visual loss. Methodology: The study utilized a mixed methods triangulation design. Clinical outcome measures were goal attainment scaling (GAS) and occupational performance (COPM) completed with qualitative interview data. Focus group interviews with participants and parents were used to evaluate the acceptability of the program. Results: GAS-rated personal goals were widely achieved and the scores of both performance and satisfaction scales of COPM improved. Overall, the rehabilitation program proved to be acceptable. Group-based rehabilitation was deemed very important and it enabled peer support. However, two-day periods of inpatient rehabilitation, proved to be too short, whereas five-day periods were considered to disturb schoolwork. Conclusions: Group-based multi-form rehabilitation for YPWVI can have a positive impact on activity and participation of the participants. The program can support independence and the achievement of rehabilitation goals. The group format was applauded for providing social support and company. The program required some structural modifications.

## 1. Introduction

Young people with visual impairment face challenges in many areas of their lives. Disabled young people may often remain children longer than their peers due to, for example, overprotective parents and helpers, a lack of assistive devices or the nature of their disability [1]. The self-esteem of young persons with visual impairment is shown to not differ from that of their sighted peers, but relationships with friends contribute to enhancing the self-esteem of adolescents with visual impairments [2]. Young persons with special needs have fewer friends and consider themselves lonely more often than those without disabilities [3,4,5,6]. Also, romantic relationships are more difficult to form [6]. Some young persons with visual impairment, especially girls, need support in their psychosocial development [4]. In addition, visually impaired young people seem to have fewer activities than their sighted peers and they seem to rely on their parents for transportation [7]. Children and young people with chronic physical conditions are 51% more likely to develop a chronic mental health condition than other children and youth [8]. Conversely, adolescents with visual impairment have reported higher rates of overall computer usage than sighted peers, due to higher usage for school work and searching for information [9]. These everyday challenges need to be tackled in their normal daily support regimen, in their education and in their rehabilitation.

These difficulties may hamper visually impaired young people’s transition to adulthood, and lead to potential difficulties in full participation as adults. Participation contributes to health and well-being, and in rehabilitation, participation is considered as the most relevant outcome [10]. The International Classification of Functioning, Disability and Health (ICF) [11]—a framework for rehabilitation practice—defines participation as personal involvement in life situations. The ICF constitutes a unified and consistent language of human functioning suitable as a reference for comparing health information [12]. The ICF brings together three perspectives—bodily, individual, and societal—and contains the following broad components: Body Function and Structures, Activities and Participation, and Environmental Factors, as well as personal factors not yet coded in the ICF. Leeuwen et al. [13] identified the most prevalent rehabilitation needs of visually impaired young adults on the Activities and Participation Component of the ICF. These needs were related to major life areas such as finding a job, mobility-related needs such as self-reliance in travelling, and needs related to communication, e.g., using communication devices and techniques. Also in this pilot study, we noticed that young persons with visual impairment faced challenges in Activities and Participation [14]. The most frequently identified challenges were related to mobility (e.g., using transportation), domestic life (e.g., doing housework), interpersonal interaction and relationships (e.g., informal social relationships such as making friends), major life areas (e.g., basic economic transactions such as using cash machine), and leisure activities. We also identified some environmental factors that can be either facilitators or barriers to participation. For example, assistive devices facilitated mobility and schoolwork but they could also be experienced as a barrier to social participation. Similarly, parental attitudes can either support or hinder a visually impaired child’s participation.

Rehabilitation is an integral component of health services, ensuring that people can realize their full functional potential in their own living and working environments [15]. In relation to the ICF, the role of rehabilitation has been described in enhancing participation [16]. Rehabilitation can address impairments, activity limitations and participation restrictions, as well as personal and environmental factors (including assistive technology) that impact on functioning [15]. However, the way in which rehabilitation is implemented and understood in real life varies and the focus is often on the impairment itself [16]. In the rehabilitation of visually impaired young people, there may be too much focus on underlying capacities instead of the different kinds of skills needed in participation [14].

In Finland, the rehabilitation of people with visual impairments is funded either by municipalities or by the Social Insurance Institution (Kela). Kela organizes medical rehabilitation services for people who have severe limitations in activity and severe restrictions in participation and pays them rehabilitation allowances. The total number of young people aged 15 to 24 in Finland in 2016 was 633,704, of whom 761 were visually impaired: 450 male, 311 female [17]. Finland is a geographically large country and even with the majority of the population concentrated in the southern region, distances between visually impaired peers may be long. This is one reason, why young visually impaired people have only few friends and often consider themselves lonely and lacking support from their visually impaired peers [14].

Kela developed and arranged (2011–2012) a new one-year group-based outpatient rehabilitation program for young people with visual impairment to support them in their everyday lives. The general aims of the program were to support young people in becoming more independent and to promote their participation. The program was developed and arranged together with the Finnish Federation of the Visually Impaired (FFVI), an advocacy organization for the blind and the partially sighted, which also provides a special rehabilitation service with social element. 

The aims of this pilot study were to describe clinical outcomes and the acceptability of the piloted program.

## 2. Materials and Methods

### 2.1. Piloted Rehabilitation Programme

A one-year, multiform, group-based rehabilitation program for visually impaired young people was arranged in two regional groups, one in the Helsinki capital area (7 participants) and one in western Finland (8 participants). 

The rehabilitation consisted of both inpatient group meetings in institutional settings and online, and individually tailored one-on-one guidance in individually selected settings (e.g., home, school or cafeteria). 

Group meetings in an institutional setting were arranged in four modules. The first module (five days) dealt with themes of interpersonal skills and life management. At this phase, a great deal of effort was put into building group cohesion among participants. The themes of the second period (two days) were social and psychological well-being, sexuality and self-determination, leisure time, and hobbies. The third module (two days) dealt with education, work and the role of the employer (in Finland disabled people employ their assistants). During the last module (five days) the participants were given practical guidance and tips for everyday life. They also received support in planning their lives and looking forward to the future. Each day included six hours of rehabilitation and three hours of other supporting activities. In addition to professional supervisors, two young persons with visual impairment acted as a part-time support team for the participants. 

One-on-one guidance in individually selected settings included interviews before and after the program and three to five meetings based on the individual needs and wishes of the young people. The focus was on the performance of practical tasks in conjunction with personal goals. Participants learned, for example, the use of public transport, made college visits and learned to use ATM machines or their assistive devices. Participants were asked to arrange one-on-one meetings with supervisors independently without their parents’ support. In addition, supervisors encouraged participants to travel independently to the meetings. 

Both real time and asynchronous tele-rehabilitation were used in the online interventions. Real time monthly group meetings (chat rooms) were built around primary themes of the rehabilitation. Some group meetings did not have a predefined theme. Supervisors provided information on an as-needed basis during chat room meetings. Participants completed tasks related to the rehabilitation themes, and in the Moodle learning platform, participants had opportunities to read more information about the themes. Supervisors supported participants with personal e-mails. Tools for this section were financed by the Finnish Ministry of Education and Culture. 

Parents had one-day group meetings twice, once in the beginning of the rehabilitation and once at the end of it. The parents program included also individual meetings in the beginning and at the end of the rehabilitation. Parents had the opportunity to reflect the strengths of their children and their own parenthood as well as to suggest their wishes for the program. The rehabilitation professionals’ peaceful presence was considered highly important. The parents’ sessions supported the agenda of the children’s rehabilitation. They had group conversations about being a parent to a growing up young person with visual impairment, and about supporting their children becoming independent. They also discussed the different roles of parenthood. Contents of their children’s rehabilitation and plans for the future were presented to the parents. Parents were provided information about the FFVI. Counsellors presented their role and services to the children and youth with visual impairments and their families. At the end of the rehabilitation parents were given advice on how to support their children’s independent living and travelling. 

### 2.2. Participants and Methods

Ethical approval for the study (following the tenets of the World Medical Association Declaration of Helsinki) was obtained from the Ethical Review Board of the Social Insurance Institution of Finland in Helsinki. All participants gave their written informed consent for participation in the study.

Inclusion criteria were age (16–22 years), visual impairment (blind, severe visual impairment or mild visual impairment), motivation for rehabilitation, lack of other severe illnesses (such as severe mental illness), lack of difficult cognitive problems and independence in daily activities (i.e., dressing, personal hygiene, eating). 

Fourteen young people with visual impairment participated in the program from the beginning to the end. They were 16–22 years of age, nine of them were male and five were female. Six were blind or had severe visual impairment and eight had mild visual impairment. All attended school—either in primary, vocational, or higher education—and took part in the rehabilitation program during their leisure time. One participant discontinued during its course. 

This study utilized a mixed methods triangulation design [18]. In this type of design qualitative and quantitative data are collected concurrently. The data were analyzed separately and then compared to corroborate the findings. Qualitative data from focus group interviews were used to assess whether the program was appropriate to the participants, and qualitative data from individual interviews were used to expand quantitative data in outcome evaluation. 

Clinical outcome measures were the rehabilitees’ occupational performance and rehabilitation goal attainment. The measurements were carried out within the rehabilitation program by the rehabilitation professionals in the beginning and the end of the program. 

The Canadian Occupational Performance Measure (COPM) was used to capture self-perceived problems in everyday activities and participation. COPM is a semi-structured interview designed to identify the activities that a person wants, needs or is expected to perform [19]. An occupational therapist trained to use the COPM interviewed the participants at the beginning of the rehabilitation program. In the first phase of the interview, participants reported the activities they found difficult to perform. Then they rated the importance of each activity on a ten-point scale, with one being not important at all and ten being extremely important. Then the participants scored their own performance and satisfaction levels on a ten-point scale with five of the most important activities and the total scores were then calculated. At the end of the rehabilitation they scored the same activities again. The new scores and the change in scores were calculated. The validity and reliability of the COPM has been widely established [20].

Goal Attainment Scaling (GAS) was used to set and score the participants’ individual goals. GAS is a method of scoring the extent to which individual goals are achieved in the course of an intervention. Participants have their own outcome measures, but standardized scoring allows for statistical analysis. At the beginning of the intervention, each participant established two to five individual goals for rehabilitation with support from professionals. Each goal was rated on a five-point scale so that the “expected outcome” fell in the middle of the scale at a score of zero. Also the levels for “somewhat less” and “much less”, “somewhat more” and “much more” were defined. At the end of the rehabilitation program, the participants evaluated their goal achievement and scored the achieved performance [21,22].

Individual interviews were used to obtain in-depth information pertaining to the experiences young persons and their parents had in terms of outcomes of the program. The young persons (*n* = 14) and their parents (*n* = 22) were interviewed by the first author of this article at the end of the rehabilitation program. The young persons were interviewed individually and their parents as couples when two parents took part in the study. Using open-ended questions modified according to the circumstances, the interviews focused on the impact of the rehabilitation program on social and independent living skills of the visually impaired participants. Additional discussions were held to describe the challenges young people experience in participation. This topic has been reported in another article about this pilot study [14]. Most of the interviews took place in the participants’ homes, while two were held in cafes and two by phone for practical reasons. The interviews were audiotaped and transcribed verbatim. Some of the young persons were quite short-spoken, so the interviews took 23 min on average (11–40 min). Interviews with parents took on average 34 minutes (15–50 min). 

Acceptability of the rehabilitation program was evaluated in focus-group interviews with the young persons (in two groups) and their parents (in two groups). The interviews focused on functionality and the importance of different modes of interventions included in the program: inpatient rehabilitation, groups, one-on-one guidance, online interventions, parents’ program and adverse effects of the program. The focus groups were run by two researchers (A-LS and TH), and each group gathered for about an hour each. Interviews were audio-recorded and transcribed verbatim. 

### 2.3. Data Analysis

The ICF was used as a conceptual framework in the data-analysis to enable the comparability between the results and health information [12]. The problems identified by the program participants by using the COPM (3–6 problems per participant) were linked with the ICF linking rules (e.g., [12]) to the corresponding ICF second-level categories chosen by the first author of this study and then checked by another researcher. The individual COPM scores from the beginning and the end of the rehabilitation were compared by use of the Wilcoxon Signed Rank test. 

The goals identified by the young people with GAS (2–5 goals per participant) were linked to the corresponding ICF second-level categories chosen by the first author of this study and then checked by another researcher. The GAS goals were incorporated into the single overall GAS T-score by applying the GAS formula, and interpreted as follows: 50, expected level of achievement; <50, performance below the expected level; >50, performance above the expected level. Means and range for the group were calculated from individual T-scores. 

The analysis of the individual interview data was carried out by the first author by means of conventional content analysis [23] with the help of the AtlasTi analysis program. By combining data from individual interviews with COPM- and GAS-measurements, it was possible to expand the understanding of outcomes of the program.

The analysis of the focus group data was carried out by the second author of this manuscript by the use of qualitative inductive content analysis. The analysis focused on the manifest and explicit level where the codes were identified within the explicit or obvious components of the data [24,25]. The codes show something important about the data in relation to the research question and they were generated from the data itself over the course of the study [24]. One sentence or meaningful data set could include one or more codes. 

The results from different data sets were combined to describe clinical outcomes, using the ICF as a conceptual framework, and the acceptability of the piloted program. 

## 3. Results 

### 3.1. Clinical Outcomes of the Rehabilitation Programme

In the COPM interviews, program participants identified 65 important problems, of which 62 were associated with the ICF component Activity and Participation [reported in more detail in 14]. They faced challenges in participation most frequently with regard to domestic life, leisure activities, major life areas, interpersonal interaction and relationships, and mobility. Scores of both performance and satisfaction scales of the COPM improved at the end of the rehabilitation program. The group level mean score in the performance of the rated activities improved from 6.1 to 7.7 and satisfaction in the rated activities improved from 6.5 to 7.9.

Each participant identified one to six goals in their rehabilitation program. In all, 39 goals were identified (mean 2.6 goals). Nearly all goals were associated with the ICF component Activity and Participation. At the end of the program, the group mean for the GAS T-score was 56.6 (range 41–72), which means that, on average, participants’ performance in their individually chosen goals was above the expected level at the end of the program. All participants progressed in some of their chosen goals. However, outcomes were individual and there were huge differences between participants. For some participants the change was related primarily to increased awareness and information on visual impairment (e.g., concerning social benefits). Overall, the participants had trained and learned many everyday skills during the program. However, many of the skills, such as more independent mobility and doing housework required long term practice and parental support before the participants were able to use the skills independently in their everyday life. Eleven out of fourteen participants could transfer at least one learned skill into everyday life. These skills were for example using public transport, transferring food from kettle to plate, frying an egg and using a bankcard.

#### 3.1.1. Domestic Life

The largest proportion of the participants’ problems (24.6%) were related to domestic life such as doing housework (*n* = 6), acquiring goods and services (*n* = 5), and preparing meals (*n* = 4). These problems were dealt with during the program, and nine participants—most of them were blind––had goals related to domestic life in their rehabilitation program (23% of the goals). Most of the goals related to acquiring good and services, such as shopping. Learning to shop is challenging especially for blind persons, as it requires learning routes and finding the courage to ask for help. One participant remarked that: *“One achievement during the program has been that I have learned to go independently to the shop and ask for help there”.* In addition to the guidance during the program, learning to shop has required parental support and guidance. Two other participants reported having practiced shopping, but not being able to do it independently at the end of the program. 

Many participants found preparing meals, and especially using a hot stove especially challenging. During the program, they had positive experiences from cooking. As one participant reported: *“Yes, we did cook during the inpatient rehabilitation period, it went well, when someone was telling me what to do, yes cooking is learnable.”* Another participant was happy about positive feedback. She said: *“I received positive feedback, for example shopping and cooking went just right, I just need to start doing them”*. However, cooking is also a skill, that requires long-term practice before it turns into everyday routine. More importantly, young participants need parental support to learn these skills. One parent said that their child had learned cooking skills during the program and would like to cook more at home: *“if only we let her. When she cooks but leaves all the cleaning to the others, it is a real mess.”*

Two participants felt it challenging to manage a household, and they had goals related to cleaning the house and washing clothes. Both of them reported having progressed in these activities during the program. 

One of the participants had the goal of moving out from her parents’ home, in which she succeeded. The supervisors discussed moving out with all participants and supported them in it. During the program, also another participant moved out on his own. Some of the parents welcomed this topic, for example one mother said: *“the course was a trigger, it gave [my daughter] the strength”*. Contrarily, another mother felt that it was too early for her child even to consider moving out, and she could not understand why this topic was even discussed in the program. 

#### 3.1.2. Major Life Areas

Nearly one fifth of the participants’ problems (18.4%, *n* = 11) were associated with major life areas, most of them were related to basic economic transactions such as using an ATM machine. Many of the participants, mainly mildly visually impaired, had a goal (28% of the goals) related to major life areas, such as school and higher education and basic economic transaction. Six participants progressed in educational matters. They had discussed professional and study choices or improved in their motivation to study. Two participants learned to use a bank-card and were able to transfer the skill into everyday life: *“At least I learned to use bank card, I use it regularly.”*

#### 3.1.3. Recreation and Leisure

A large proportion (23%, *n* = 15) of the participants’ problems were associated with recreation and leisure. This included problems related to meeting friends, participating in sports and playing games. However, only two participants had goals related to these in their rehabilitation program. One of them got active in camp activities offered by FFVI.

#### 3.1.4. Interpersonal Interactions and Relationships

Even if only three participants had goals (8% of the goals) associated with interpersonal interactions and relationships, all participants said that they were happy to meet other visually impaired young persons during the program. Group based rehabilitation was a good platform to learn interaction skills with peers. One participant was happy about having learned to ask strangers for help during the program. “*My goal was to get more courage, social skills, to get the courage to ask strangers for help, and that I could go talk to others at school. I have progressed in these.”*

One of the participants told that: “I do not usually get along in groups, here it has gone surprisingly well”. Another participant said: “It is good to find out that I am not the only one.” And a third one: “In this program it is so different to be with other visually impaired, everything is better.” 

Some participants formed new friendships with each other during the program, but only few of them reported to keep in touch after the program. Many parents had the same question at the end of the program: how to tackle with loneliness?

#### 3.1.5. Communication

Two participants had communication-related goals. One of them wanted to learn discussion skills, as he considered it important to learn how to initiate discussions. At the end of the program, he thought that he had progressed according to his plan. Another participant wanted to learn to use computer-based communication techniques: *“It would then be easier in high school”.*

#### 3.1.6. Mobility 

Only few participants had mobility-related goals, but supervisors required each one of them to try independent mobility and to use public transport independently when they travelled to the inpatient module of the rehabilitation. Some participants reported that their ability to use public transport had improved during the program, when earlier they had primarily taken a taxi.

Especially parents applauded this: “*Along with the program came independent mobility. [Our daughter] has travelled to program meetings independently. She was forced to do these tasks, but I believe that this has been good for her.*”

#### 3.1.7. Environmental Support and Assistive Devices

Overall, the participants reported that the program supported them in looking after themselves and gave them visions about more independent living. Support from and relationships with supervisors and peers, and an encouraging attitude during the program gave a boost to the participants. Most of them became more active and were encouraged to look after themselves. *“I have learned to take more responsibility of my own life”.*

Many participants reported the importance of their parents in encouraging and teaching independent living skills. In some families, parents allowed participants to practice their skills: *“During the program I learned to fry eggs, and now I have been able to do so at home, and my mother lets me.”* Still, many parents do the tasks automatically for their children. Two participants considered their parents overprotective. One of them thought that she did get some help with this during the program: *“In a way this program has helped my mother more than me, she has learned to trust me more. She has learned to trust, to not always worry, that she always does so naturally.”*

At the beginning of the program, some participants either had improper assistive devices or were not able or willing to use proper devices efficiently. During the program, seven participants were either purchased new assistive devices or were referred to assistive device assessment. They also practiced to use a white cane and computers. 

### 3.2. Acceptability of the Rehabilitation Programme

#### 3.2.1. Length of the Program

Both the program participants and their parents considered the length of the rehabilitation program suitable. One year was a suitable time frame to work toward their goal achievement. 

#### 3.2.2. Inpatient Rehabilitation Periods

Five-day modules made it possible to focus on rehabilitation topics with time and were easy to cope with. Most participants considered the two-day inpatient module to be too short with too much time spent in the program. The participants considered the contents of the rehabilitation modules to be worthwhile.

Leisure time during inpatient periods was very important for the participants as it gave them the opportunity to spend time with their peers. Participants considered having slightly older peers as role models supporting them during the inpatient module to be an important component of the program that set a good example. 

#### 3.2.3. Group-Based Rehabilitation 

Young people with visual impairment considered group-based rehabilitation to be very important. The group provided the opportunity to get to know other visually impaired young people, learn from them, make friends and get peer support. The participants described the atmosphere in the groups as good and accepting. The size of the group (seven people) was considered good as it enabled both getting to know other people and individual participation. One participant said: *“It has been good to have two groups. The group is small enough to learn to know each other…In a bigger group it is more difficult to get your voice heard. And those practical tasks, such as cooking, are easier in smaller group. Everyone could participate.”* However, the two groups were different from each other. One group also spent leisure time together during the inpatient rehabilitation periods, whereas the other divided into smaller subgroups. The participants reported that it was unproblematic and nice to have both blind and mildly visually impaired participants in the same group: *“It is only good to have both, the activity is not dependent on if you have sight or not, we all are similar, however.”* Also, most parents considered this arrangement good, as it provided an opportunity for mildly visually impaired participants to help the blind participants, which might raise their self-esteem. On the other hand, blind participants were able to show the mildly visually impaired participants that you can manage even with severe challenges. On the other hand, some parents thought that it would be better to have a more homogenous group, because it would allow focusing on disability-centered problems. For example, asking for help is different for participants whose visual impairment is not visible than for those who are blind. Participants and parents highlighted the importance of not making changes to groups during the rehabilitation process. 

#### 3.2.4. Parents’ Program

Parents considered their program important, but too short. Time was spent largely on providing them with information and the time left over was not sufficient for discussions. Especially parents would have liked to have some spare time together. One parent considered getting much new information in such a short time as too heavy. Also, the time between parents’ meetings was lengthy and some of the parents proposed one extra day for parents in the middle of the program. Parents who were new to the topic of visual impairment considered the opportunity for peer support to be especially important. Some parents would have preferred more support during the program: *“I would have liked encouragement with how to guide the child, to get support in how to give her more space.”*

#### 3.2.5. One-to-One Guidance

Both the project participants and their parents considered one-on-one guidance to be useful and supportive. One-on-one guidance was implemented in real-life environments and situations that were important and meaningful for the participants and supported their rehabilitation goals. Performing practical tasks with a connection to personal goals with the support of the supervisors prompted true pleasure and a sense of goal achievement. Many of the young participants considered the support by professional supervisors to be more helpful than the support given by their parents. It is easier to accept support and feedback from an outsider.

#### 3.2.6. Online Interventions

Online rehabilitation divided opinions, with girls being more positive and boys more critical. Some participants took part in all of the real time meetings and gained a lot from the asynchronous tasks, while some participated in only a few meetings and did not read or do any of the tasks in the learning platform. Most participants described that chat rooms were important places to meet other young people, when face-to-face meetings were impossible to arrange. The findings suggest that online rehabilitation supported direct communication between young people and interaction through the rehabilitation professionals decreased during the rehabilitation program. Furthermore, some participants and parents reported that online rehabilitation developed participants’ information and communication skills.

Many participants reported that they had major difficulties in coordinating their school schedule with participating in the online chat room. Participation in the online interventions was disrupted also due to technical issues. Some participants experienced the online rehabilitation as unhelpful, for example the contents of the interventions were not relevant to them. One participant reported: *”During one-to-one guidance we can focus on the place where we are and visit different places, in the chat we focus more on ideology.”* Parents expressed that they would have preferred more information about the online intervention. 

#### 3.2.7. Adverse Effects

No adverse effects were reported other than inpatient rehabilitation modules during school weeks having disturbed studying and students had to make up for all from school. However, even with the need to make up for missed time in school, the participants were not willing to have institutional rehabilitation modules during school holidays. Additionally, parents expressed concerns about their children managing both school and rehabilitation at the same time because school is very time-consuming for them. 

## 4. Discussion

Results of this study show that a multiform, group-based rehabilitation program for young people with visual impairment is beneficial in promoting activities and participation. Due to, for example, parental overprotection and a lack of activities, it is beneficial to provide them extra support on their path towards independence. Some of the participants in this study will never be fully independent, but will live with interdependence instead, in which a person does some things with assistance and others without [26]. In these cases, activity with assistance may be a realistic goal for many visually impaired youths, and it is important to explain this to them instead of focusing only on independence [26]. 

Group-based rehabilitation proved to be especially important for the young participants. Like the participants in this study, young persons with disabilities consider themselves lonely more often than those without disability [4,5]. Social support, especially the support of peers, is important to adolescents with visual impairments [27] and may contribute positively to their self-esteem [2]. Group meetings, both face-to-face and online, provided participants the opportunity to meet their peers, which is important especially in sparsely populated countries or areas. Indeed, some of the participants’ experiences identified in this study relate to the therapeutic factors of group therapy as described by Yalom [28]. Peers and acceptance relate to Yalom’s universality: The realization that one’s concerns or problems are not unique but experienced by others as well. Furthermore, acceptance relates to Yalom’s concept of group cohesiveness: a trustworthy, warm, empathetic, understanding and accepting atmosphere is therapeutic. Advice and encouragement relate to Yalom’s altruism because giving advice and encouraging each other give an opportunity to be of help to others.

Furthermore, peer support from more experienced young persons with visual impairment is important, as this way young people can better understand their potential and their parents can understand that having expectations for their children is both realistic and a critical part of their child’s development [29]. Peer support from more experienced young persons may, for example, improve participants’ negative attitudes towards assistive devices, which often have a crucial role in enabling activity and preventing underachievement.

Interestingly, participants were divided on the online components of the intervention and criticized them widely. Young people tend to spend their social lives on the Internet—according to a Finnish study, 16% of youths aged 13–16 spend more time with their friends on the Internet than face-to-face and 41% have good friends with Internet contact only [30]. Young people participate in online activities and social media most commonly for friendship or interest-driven purposes. Interest-driven means that the time spent online is focused on a certain interest, activity or hobby [31]. Criticism about the online segment of rehabilitation might be due to young people finding other social media or Internet forums more motivational than tasks and information presented in the learning platform. A potential explanation concerning the criticism of the online activities might be the lack of parental involvement or technical problems with the learning platform. According to Yu et al. [32] and Raghavendra et al. [33], young people with health-related problems value the social and emotional support from families and close friends and technical support on the use of the Internet as a venue for social networking and sharing feelings and experiences with others. 

After the completion of this program, the use of tele-rehabilitation has increased widely. As of yet, there seems to be only little evidence about tele-rehabilitation among visually impaired people [34]. However, low vision tele-rehabilitation has proved to be feasible and acceptable [35]. A recently published study provides evidence of the effectiveness of a home rehabilitation intervention for visually impaired adults by showing an improvement of visual functions [36]. The focus of the intervention was on underlying capacities, whereas in this program it was on activity and participation. It may be that a broad content area brings an extra challenge into online interventions. 

The results of this study show that the program succeeded to focus not on underlying capacities but instead on the different kinds of skills needed in participation. Concrete action and being engaged in something that is meaningful and important for the individual concerned is a way for people to become what they want and have the potential to become [37]. Through the execution of tasks and activities, a person gets involved in life situations, which means that they participate [11]. How young persons with a visual impairment negotiate socially within their life contexts is a determining factor in the ultimate level of independence in their lives [38]. For young people with visual impairment, concrete and independent action supported by the supervisors improves their potential to participate. On the one hand, it gives them real-life feedback regarding their abilities that they tend to estimate better than their parents [14,39], and on the one hand, they may show their potential to their parents who tend to underestimate their capabilities. Still, for many, transferring the potential, for example newly learned skills, into real life requires long-term support from both supervisors and parents.

The program for parents was too limited in this program. Parents play a crucial role in the socialization process of young people with visual impairment [29]. There are individual differences among parents with respect to their acceptance, involvement, and understanding of the social development process, and parents may have feelings of isolation due to their visually impaired child and a need for practical information sharing. Therefore, they need, in addition to professional support, peer support from other parents. 

Overall, the program is appropriate for young people with visual impairments. The program still requires some structural modifications. Possibly two or three 3–4-day inpatient rehabilitation modules would produce a similar outcome and disturb studying less. Online intervention components could be limited to informal discussions. Parents need a more intense program that could partly take place online.

The strength of the study lies in its mixed methods triangulation design describing how visually impaired young people and their parent report the outcomes and acceptability of the piloted program. Subjective COPM and GAS measures were used in addition to the interviews as the method of data collection. The use of a mixed-method design has both theoretical and practical strengths by involving stakeholders in the research process and by enhancing context sensitivity in line with models such as the ICF, that leads to the potential ecological and external validity of research [40].Thus the research approach is philosophically and methodologically congruent with the study [41]. The primary limitation of the study is the small sample size, which is limited in its statistical power. However, significant improvements after intervention for total performance self-ratings on both the COPM and GAS T-scores and interview data support the positive outcome. Involving visually impaired young people in the evaluation of outcomes and acceptability of the program helps represent their interests and ensures that young people’s voices have been heard. The use of the ICF as the conceptual framework in the data analysis and the results of the study increases the comparability of information, and makes the results available in a consistent manner to clinicians and decision makers [12].

The group exceeded a mean normalized T-score of 50 on GAS, indicating that, on average, goals were above the expected level. On the other hand, this—as well as the broad range of the T-scores—may show difficulties in the goal-setting process, leading participants to set goals that were either too easy or too difficult to achieve. 

Originally, the COPM and GAS were intended to be used in a combination in this intervention. The COPM was first used by an external occupational therapist to identify the problems of young persons after which GAS was used by the group supervisors to define the goals for implementation. However, the ICF linking of the COPM problems and GAS goals showed that the setting of GAS goals was not based on the COPM assessment. The main reason for this was lack of cooperation between professionals. On the one hand, the group supervisors had thorough information on the participants from their medical background data and personal interviews that may have had impact on setting of GAS goals. Previous research [42] has shown that a combined use of the COPM and GAS—although time-consuming—results in goals that are perceived almost unanimously as client centered. Furthermore, the combined use has been shown [42] to enable the subjective and objective demonstration of goal achievement, thereby supporting the clinical utility and treatment validity of the combined use of these tools. On the other hand, there is evidence that the use of the COPM did not grasp the challenges related to body functions or environmental factors with visually impaired young people, which highlights how critical it is, to complete the use of the COPM with an evaluation of factors that may have an impact on participation [15]. 

## 5. Conclusions

A multiform, group-based rehabilitation program for young people with visual impairment is beneficial in promoting activities and participation. It is necessary in a rehabilitation program to focus on the goals and areas that the participants themselves consider important. The crucial elements of the program are practical support and focus on action within real life contexts, both individual and group interventions, peer support, and parental support. 

Difficulties in activities and participation, as well as restricted environmental factors of young people with visual impairment should be recognized in all health care, social care and educational settings to promote full participation and wellbeing of the young.
